# Tradeoffs between chilling and forcing in satisfying dormancy requirements for Pacific Northwest tree species

**DOI:** 10.3389/fpls.2015.00120

**Published:** 2015-03-03

**Authors:** Constance A. Harrington, Peter J. Gould

**Affiliations:** ^1^United States Department of Agriculture Forest Service, Pacific Northwest Research StationOlympia, WA, USA; ^2^Washington Department of Natural ResourcesOlympia, WA, USA

**Keywords:** chilling, forcing, dormancy, possibility line, budburst, parallel model

## Abstract

Many temperate and boreal tree species have a chilling requirement, that is, they need to experience cold temperatures during fall and winter to burst bud normally in the spring. Results from trials with 11 Pacific Northwest tree species are consistent with the concept that plants can accumulate both chilling and forcing units simultaneously during the dormant season and they exhibit a tradeoff between amount of forcing and chilling. That is, the parallel model of chilling and forcing was effective in predicting budburst and well chilled plants require less forcing for bud burst than plants which have received less chilling. Genotypes differed in the shape of the possibility line which describes the quantitative tradeoff between chilling and forcing units. Plants which have an obligate chilling requirement (Douglas-fir, western hemlock, western larch, pines, and true firs) and received no or very low levels of chilling did not burst bud normally even with long photoperiods. Pacific madrone and western redcedar benefited from chilling in terms of requiring less forcing to promote bud burst but many plants burst bud normally without chilling. Equations predicting budburst were developed for each species in our trials for a portion of western North America under current climatic conditions and for 2080. Mean winter temperature was predicted to increase 3.2–5.5°C and this change resulted in earlier predicted budburst for Douglas-fir throughout much of our study area (up to 74 days earlier) but later budburst in some southern portions of its current range (up to 48 days later) as insufficient chilling is predicted to occur. Other species all had earlier predicted dates of budburst by 2080 than currently. Recent warming trends have resulted in earlier budburst for some woody plant species; however, the substantial winter warming predicted by some climate models will reduce future chilling in some locations such that budburst will not consistently occur earlier.

## Introduction

Many woody plant species in the temperate and boreal regions have a chilling requirement to ensure that budburst occurs during the optimum time period in the spring (c.f., recent reviews by Rohde and Bhalero, 2007; Campoy et al., 2011). The chilling requirement, or experience of cool temperatures by plant buds (or apical meristems since some species that do not set bud may also have a chilling requirement), promotes rapid bud burst (or apical growth) in the spring. This is of particular interest as winter temperatures are predicted to increase with global warming (IPCC, 2013) and changes in phenology have already been observed (Ahas et al., 2002; Jeong et al., 2011). Although earlier budburst may occur with warmer winter and spring conditions, the effects will not be the same for all species or under all conditions. Woody plants generally experience a reduction in forcing (or thermal time) with increased chilling (Cannell and Smith, 1983, 1986; Murray et al., 1989) but the magnitude of the effect differs with species. In addition, since each location has its own climate and species (or genotypes) differ in their chilling requirements, the effects of warming may advance or retard budburst (or result in atypical development) depending on how the winter environment satisfies the plant's chilling requirement (Chandler et al., 1937; Weinberger, 1967; Cannell and Smith, 1983, 1986). More recent studies have also developed or used existing models of dormancy to predict future timing of bud burst associated with climate change (c.f., Murray et al., 1994; Cumming and Burton, 1996; Schwartz and Hanes, 2010). Other studies on dormancy have focused on describing the phenomenon with the goal of managing bare-root or containerized nursery plants in cold storage (van den Driessche, 1977) or selecting horticultural varieties for specific climates (Saure, 1985).

The effect of winter temperatures on dormancy has long been studied (e.g., Molisch, 1906 cited in Sarvas, 1974) but the physiological mechanisms involved in perceiving and responding to chilling are still not well understood. Several recent studies have documented changes in gene expression before, during, and after vegetative and reproductive budburst (Leida et al., 2010; Ubi et al., 2010). Although many studies of dormancy have been published, few have proposed specific mechanisms that could be involved in plant sensing and “remembering” environmental conditions (Sung and Amasino, 2005). One exception is the work with birch (*Betula pubescens*) and *Populus* by Rinne and colleagues in which they proposed that short days promote production of 1,3-β-D-glucanase which blocks plasmodesmatal transfer to buds and then chilling removes glucan plugs from plasmodesmal channels which then permits movement of signals promoting shoot growth to their targets in the shoot apex (Rinne et al., 2001, 2011). On the other hand, mitotic activity in the apical bud of three hardwood species was more closely related to air temperature than a measure of dormancy such as days to budburst (Calmé et al., 1994). Since mitotic activity would be an indication of cell division which is required for budburst, their observation may imply that signals are being transmitted to the buds in the fall and winter and thus, that if plugs are present, they are not preventing the movement of chemical signals. While these studies do not directly contradict each other, it appears that the range of physiological mechanisms associated with the onset and release of dormancy are not completely understood and it is possible that more than one mechanism could be involved in promoting and releasing dormancy.

The timing of budburst or breaking of dormancy can be modeled in many ways (Kramer, 1994); the most common models are sequential, where it is assumed that a chilling requirement is satisfied before forcing is effective, or parallel, where it is assumed that both chilling and forcing are perceived by dormant plants. Many other types of dormancy models have been proposed and tested including models based on physiological assumptions about plant development (Hänninen, 1995; Hänninen and Kramer, 2007). Recent work (Harrington et al., 2010) suggested that the parallel or concurrent model of dormancy was appropriate for Douglas-fir (*Pseudotsuga menziesii*). This was initially proposed with data from a limited number of families and then supported with additional data from a larger number of families from a wider geographic range (Gould et al., 2011).

Many researchers have suggested that genotypes differ in the temperatures which are effective in chilling or forcing (c.f., review by Romberger, 1963). However, based on evidence of the relative efficacy of different temperatures in releasing bud dormancy or promoting vernalization for several species it is also possible that the basic effects of specific temperatures in promoting chilling or forcing are the same for many species (Harrington et al., 2010). This is consistent with the models developed by others in which chilling and forcing (or thermal time) is defined in the same way for all species being considered (c.f., Murray et al., 1989). This study presents data from 11 Pacific Northwest tree species to further test the concepts that: (1) the parallel model of dormancy is effective in predicting budburst and (2) the tradeoffs between chilling and forcing in permitting budburst (the possibility lines) are similar but not identical across species or genotypes. The calculation of possibility lines for species or genotypes allows modeling of changes in date of budburst under different climate scenarios. This permits identification of species and landscape positions which will be most sensitive to future changes in winter temperatures.

## Materials and methods

Seedlings from 11 Pacific Northwest tree species were selected for use in dormancy trials (Table 1). A minimum of 100 seedlings per conifer seed source were used each year; the conifer seedlings other than Douglas-fir and Pacific madrone were purchased were purchased as described below. The Douglas-fir seedlings (three open-pollinated families) were grown in our facility in Olympia, Washington; data from these seedlings was combined with data from previous trials with Douglas-fir (Gould et al., 2011). The Pacific madrone seedlings were obtained from the Washington State University Research Station in Puyallup, Washington. The madrone seedlings came from six geographically diverse sources; they were divided into a mild climate group (seed sources from coastal California) and a colder group (sources from Vancouver Island, British Columbia, northwest Washington, and high elevation in California). Seedlings for the other nine species were purchased from the Washington Department of Natural Resources Lt. Mike Webster Nursery; one or two seed lots per species were purchased each year. Seedlings were 1–3 years old and had been grown in pots, styroblocks or bareroot nursery beds. All seedlings were transplanted in the spring prior to the trials into 7.6-l pots filled with a commercial potting mix composed of peat moss, perlite, and a controlled release fertilizer source. There were 1–3 seedlings per pot; each pot contained only one species due to differences in seedling sizes and expected growth rates. Seedlings were grown on tables in an open-air facility until the fall treatments commenced.

**Table 1:** **Description of seedlings used in dormancy trials**.

**Species**	**Common name**	**Years of data**	**Nursery lots (Sold as suitable for or collected from)**
*Abies amabilis*	Pacific silver fir	1	PC10-479 (Elochman 0–457 m)
*Abies grandis*	Grand fir	2	PU10-588 (Upper Chehalis 0 = 305 m)
*Abies procera*	Noble fir	2	PU09-375 (Lewis, 0–1219 m)
*Arbutus menziesii*	Pacific madrone	2	Warm group: LA5 (Los Altos, CA); LA13 (Los Altos, CA); SA7 (Santa Cruz, CA). Cool group: BC6 (Nanaimo, BC); FB3 (Anacortes, WA); SN1 (Sierra Nevada, CA)
*Larix laricina*	Western larch	2	PU10-605 (Pend Oreille, 610–914 m)
*Thuja plicata*	Western redcedar	2	PU10-602 (Twin Harbors, 0–610 m); PU10-611 (Puget Sound, 0–610 m); OL10-510 (Puget Sound 610–1219 m)
*Tsuga heterophylla*	Western hemlock	2	OL10-507 (Hoh 366–732 m); WA coast 0–610 m)
*Pinus contorta*	Lodgepole pine	2	PU09-391 (Twin Harbors, 0–305 m); PU10-590 (Cowlitz 914–1210 m)
*Pinus monticola*	Western white pine	2	SE10-489 (Lower Columbia, 0–2743 m); SP10-486 (Lower Columbia 0–2743 m)
*Pinus ponderosa*	Ponderosa pine	1	NE10-533 (Kettle, 914–1219 m)
*Pseudotsuga menziesii*	Douglas-fir	2	3 o.p. families: 44.623°N, −123.546°W, 244 m; 47.181°N, −123.560°W, 61 m; 43.027°N, −122.871°W, 877 m; added to data from (Gould et al., 2011) with > 100 o.p. families

*Nursery lots were bulk collections; o.p. lots were seeds collected from open-pollinated cones collected from one tree*.

Treatments were designed to create a range of chilling and forcing units; there were 18–24 seedlings per plant lot in each treatment. In early fall, pots were randomly assigned to one of 4–6 temperature treatments each year (Table 2). All plant materials were tested under treatments Warm, Warm plus Cool Interruption, Ambient plus Warm 2, and Ambient. The Ambient plus Warm1 and the Cold treatments were used for some sources and in 1 year. The Cold treatment involved sinking pots into the ground so that plants would experience soil and air temperatures similar to the environment of planted or natural seedlings. The Ambient treatment placed seedlings on benches in a covered lath house (sides of the building open for exchange of ambient air). The greenhouse used for the Warm treatment and the Ambient treatments with Warm 1 or Warm 2 was set for a minimum temperature 15°C; this temperature threshold was not met 2–3 days each year due to brief power outages. The movement of seedlings in the Warm plus Cool Interruption treatment to the ambient environment was delayed by a few days if the temperatures in the outdoor environment were forecasted to be below −3°C; this was done to avoid damaging seedlings which may have not been cold hardy. Ambient temperatures from Nov 1 thru March 31 averaged 4.0°C and 4.8°C in the 2 years (2011–2012 and 2012–2013). The Warm treatment averaged 15.1°C, the Cold treatment 2.5°C.

**Table 2:** **Description of treatments used in dormancy trials with northwestern species**.

**Treatment name**	**Days/week in 15°C greenhouse**	**Days/week outside**	**Days/week in 18°C growth chamber**	**Timing**
Warm	7	0	0	All months
Warm plus cool interruption	6	1	0	All months
Ambient plus Warm 1	1	6	0	All months
Ambient plus Warm2	2	5	0	All months
Ambient	0	7	0	All months
Cold	0	7, high elevation	0	All months
Force Early	0	3	4	6 weeks Nov–Dec
Force Late	0	3	4	6 weeks Jan–Feb

*Days per week outside refers to ambient conditions at Olympia, WA, USA (elevation 65 m except for the Cold treatment where plants overwintered at a site at 885 m)*.

The forcing treatments (Force Early and Force Late) were conducted in Percival growth chambers (Models PGC 9/2 or PGC 15) set at 18°C. The chambers used a mixture of incandescent and florescent bulbs to provide lighting. All treatments experienced natural photoperiod (the chambers were programmed to shift photoperiod daily). Continuously recording temperature monitors (temperature or temperature/relative humidity Hobos from Onset Computer Corp. set to record the temperature at 30-min. recording intervals) were placed in each environment at approximately the mean height of seedling buds.

At the beginning of each trial seedlings were tagged, measured for height and diameter, and atypical conditions noted. Because western redcedar does not form vegetative buds, the terminal shoot on each seedling was marked approximately 1 cm back from the tip using a broad-tip felt pen with permanent ink; this was done so repeated measurements could be used to determine when shoot growth had resumed in the spring.

Seedlings were checked weekly for budburst (or twice weekly during the most rapid period of budburst); budburst was determined to occur when the bud scales had parted sufficiently to see green leaf tissue or when the tip of western redcedar was 2 cm from the bottom of the mark made in the fall (i.e., when approximately 1 cm of growth had occurred). Pine species were also coded for shoot elongation which occurred prior to needle emergence. We later determined that the shoots of some seedlings in the low chilling treatments initially coded as “bud burst” did not continue to elongate past the point of parting the bud scales; these seedlings were re-coded to indicate normal budburst had not occurred.

**Figure 1:**
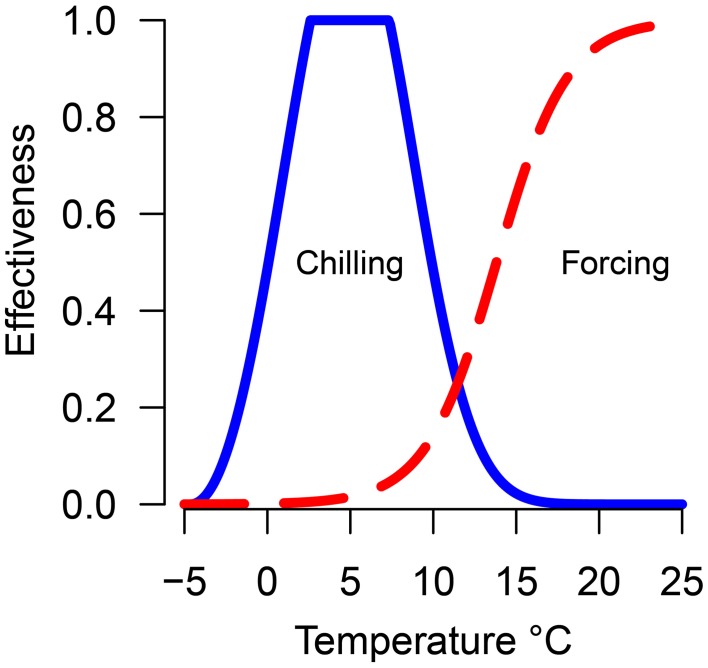
**Predicted efficacy of temperature in promoting chilling (solid blue line) or forcing (dashed red line)**.

Chilling units and forcing units were calculated daily for each treatment using the previously calculated equations relating temperature to chilling or forcing effectiveness (Harrington et al., 2010) (Figure 1). Hours at each temperature were multiplied by the effectiveness value. The accumulation of both chilling and forcing begin November 1 and continued until budburst. For each species/seed source/temperature-treatment combination, a point was plotted to indicate the accumulated chilling and forcing hours at the date of 50% budburst. The date of 50% budburst was chosen to provide a central response for populations rather than focusing on the first or last plants to burst bud as those events may be outliers from the general responses. Lines (named possibility lines in Harrington et al., 2010) were fit thru or below the points representing each treatment to indicate conditions where budburst was possible; points above or to the right of the line are possible combinations of chilling and forcing which will result in budburst while points below or to the left of the line are conditions which will not result in budburst. Data for each species were plotted with points from each seed source and trial plotted with different plotting symbols. Except for Pacific madrone and western redcedar (described under results) the data points were combined for each species to fit possibility lines. The possibility-line model form was forcing = a_0_ + exp (a_1_ + a_2_· chilling). Models were fit using non-linear least-squares regression with the nls function in the R statistical software (Bates and Watts, 1988; R Core Team, 2013). Some species/treatment combinations did not result in 50% of the seedlings bursting bud; for these combinations the lines were adjusted if needed to result in a line which did not cross the forcing hours axis (e.g., weighed regression was used to adjust the left end of the line upward to become asymptotic to the y-axis, thus implying the species or genotype had an obligate chilling requirement). Data on shoot elongation in pine prior to budburst was used to inform our decision on adjusting line placement for the pines at low levels of chilling. Since the line for Douglas-fir is based on the most number of years and conditions, we compared results from other species to the line for Douglas-fir.

To project changes in the timing of budburst with climate change, the average date of budburst under the current climate and predicted climate in 2080 was calculated at the locations of 304 weather stations in western Washington and Oregon. Hourly temperature records for 8 of the past 12 years (to compensate for missing records at some stations in some years) were used with the possibility line models for each species to calculate the date of budburst. The projected date of budburst in 2080 was calculated by determining the predicted change in monthly temperature at each weather station location between the current climate and predicted climate in 2080 based on the consensus model using the A2 emission scenario from ClimateWNA (Wang et al., 2012). Climate predictions were downscaled to the weather station locations using the ClimateWNA model. The changes in monthly mean temperature were added to the contemporary hourly climate data to simulate hourly data in 2080. The predicted average increase in temperature across all weather stations was 3.9°C with a range from 1.6 to 6.1°C (range across stations for each month). The possibility line models were then applied to the future climate data to estimate budburst in 2080. The results were interpolated across the Pacific Northwest using a thin-plate spline model based on the latitude, longitude, and elevations of the weather stations (Nychka et al., 2014). The results were displayed on maps clipped to the outline of the range for each species within western California, Oregon, Washington (USA) and southern Vancouver Island, British Columbia (Canada). Our landscape predictions resulted in large differences between current and predicted date of budburst with the largest differences observed for Douglas-fir. To provide additional verification of the reasonableness of these predictions we plotted date of bud burst against mean winter temperature for 3 years on nine sites.

## Results

### Seedling responses

**Figure 2:**
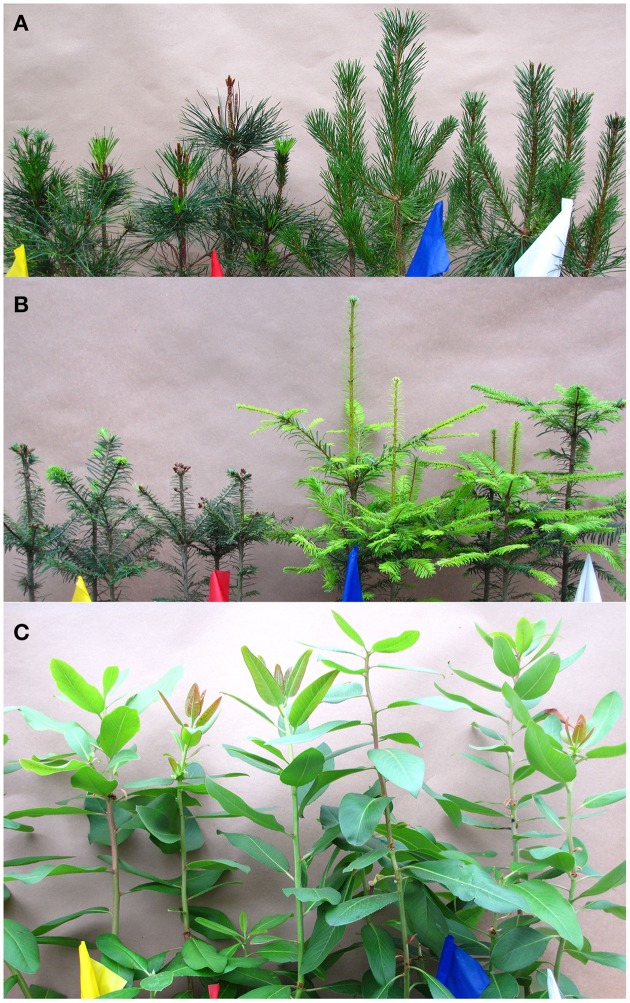
**Pictures taken in August 2013 of seedlings receiving a range of chilling and forcing hours during the 2012–2013 fall-winter-spring seasons**. The colored flags indicate four temperature treatments from warmest (left) to coolest (right): yellow flag, Warm (in greenhouse continuously); red flag, Warm plus Cool Interruption, (greenhouse with 1 day/week outdoors); blue flag, Ambient plus Warm1 (outdoors with 1 day/week in greenhouse), and white flag, Ambient (outdoor conditions only). Shown from top to bottom are: **(A)** lodgepole pine, **(B)** grand fir, and **(C)** Pacific madrone.

Seedling appearance was normal for Pacific madrone, western redcedar, and western hemlock in all treatments. For other species, low levels of chilling resulted in elongation without needle development (pines), lack of budburst or abnormal growth after budburst (true firs, Douglas-fir, pines), and change in terminal leader with many buds not bursting (Figure 2). Plants exhibiting abnormal appearance in the spring remained that way throughout the subsequent growing season.

**Figure 3:**
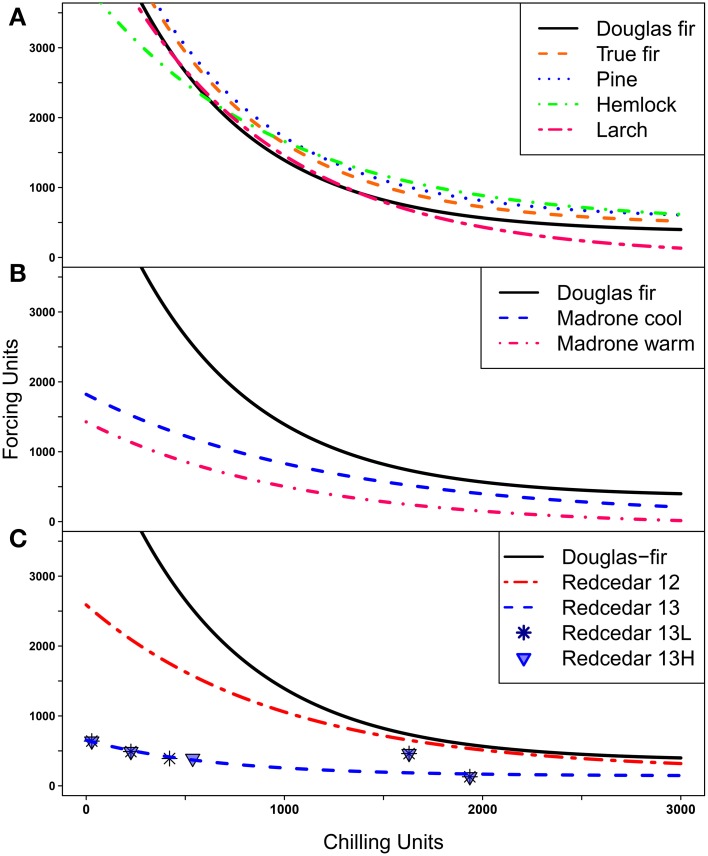
**Possibility lines separate combinations of chilling and forcing; points above or to the right of the lines indicate combinations where budburst is possible, points below or to the left of the lines indicate combinations where budburst is not possible. (A)** Possibility lines for Douglas-fir, three species of true fir combined, three species of pine combined, western hemlock, and western larch. **(B)** Possibility lines for two groups of Pacific madrone (three seed sources from cool and three seed sources from warm climates) compared to the line for Douglas-fir; **(C)** Possibility lines for western redcedar based on trials from 2012 (12) and 2013(13) with the line for Douglas-fir shown for comparision. Points for the 2013 trial are plotted separately for the low elevation (13L) and the high elevation (13H) source.

The responses for Douglas-fir in this trial were consistent with the possibility line from previous seedling trials (Harrington et al., 2010) and with dates of bud burst in field trials (data not shown). We were able to fit possibility lines for all species or groups of species that were similar to the possibility lines previously calculated for Douglas-fir (Harrington et al., 2010; Gould et al., 2011), that is, the lines developed for other species all exhibited the tradeoff between chilling and forcing for budburst (Figure 3). The Ambient conditions during the 6-week periods when the seedlings were in an 18°C chamber for 4 days per week were not identical for seedlings in the Force Early (November–December) and Force Late (January–February) treatments; however, the point for each species were close to the possibility line and provided no indication that the timing of the additional forcing changed the plant responses (data not shown).

The possibility line for western larch was quite similar to that for Douglas-fir at low levels of chilling (Figure 3A); this implies larch also has an obligate chilling requirement but will burst bud at low (but not zero) levels of chilling if large numbers of forcing units are present. At the other end of the curve where plants have experienced high levels of chilling, very little forcing is needed for budburst. Western larch was typically the first species to burst bud at high levels of chilling.

None of the three true fir (*Abies*) species burst bud at low levels of chilling, thus, indicating they all had obligate chilling requirements. The data for the three species did not differ from each other at moderate or high levels of chilling so only one line was fit for these species (Figure 3A). The true firs required more forcing to promote bud burst at high levels of chilling than was true for Douglas-fir so the possibility line for true fir was fit above the line for Douglas-fir.

The three pine species also required chilling for budburst (i.e., had obligate chilling requirements). Since the data points were very similar for the three pine species at high levels of chilling and we had relatively little data on budburst for pine at low levels of chilling, we fit the possibility line for the three pines together (Figure 3A). However, ponderosa pine had only one data point below 1000 chilling hours (compared to three for western white pine), thus, the pine possibility line may not accurately represent the response of these pine species at low chilling. At high levels of chilling the pines generally required more forcing for bud burst than was observed for Douglas-fir so the pine possibility line was consistently higher than the line for Douglas-fir.

The possibility line for western hemlock crossed the line for Douglas-fir (Figure 3A). This implies that at low levels of chilling western hemlock required less forcing for budburst than Douglas-fir but on the other hand, at high levels of chilling western hemlock required more forcing for budburst than Douglas-fir. Thus, in the cool treatments Douglas-fir burst bud sooner than western hemlock while in the warm treatments the reverse was true.

Pacific madrone and western redcedar were the first species to burst bud under no, low or moderate chilling. For these two species, the shape of the possibility lines (Figures 3B,C) at low chilling (i.e., the flatter lines) implies that the genotypes tested have no or limited chilling requirements; that is the possibility lines for those species will intersect the y axis when the plants have received no chilling hours. We had two groups of madrone genotypes, the “warm” group had a lower possibility line (lower intercept on the y axis) than the “cool” group but almost all seedlings in both groups burst bud without chilling (Figure 3B). For western redcedar the two genotypes (lower and higher elevation seed sources) tested in 2013 had almost identical responses, however, the combined line fit for two genotypes from 2013 data was substantially lower than the line based on previous data (Figure 3C). This was the only species where the data from multiple years did not appear to be fitting the same relationship. To be conservative, we used the 2012 possibility line for redcedar for our landscape analyses.

### Landscape responses

**Figure 4:**
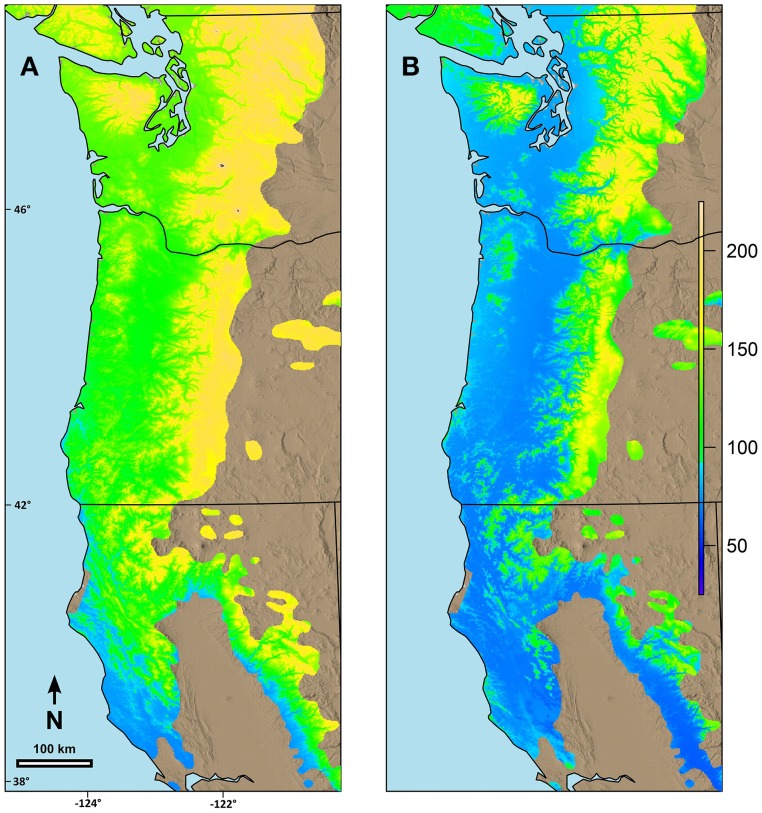
**Predicted date of terminal budburst for Douglas-fir under: (A) current climatic conditions and (B) climatic conditions in 2080 based on the A2 scenario in ClimateWNA**. The colors reflect number of days since the beginning of the year (1 = Jan 1). Numbers on the left and the bottom of the map refer to latitude (north) and longitude (west).

**Figure 5:**
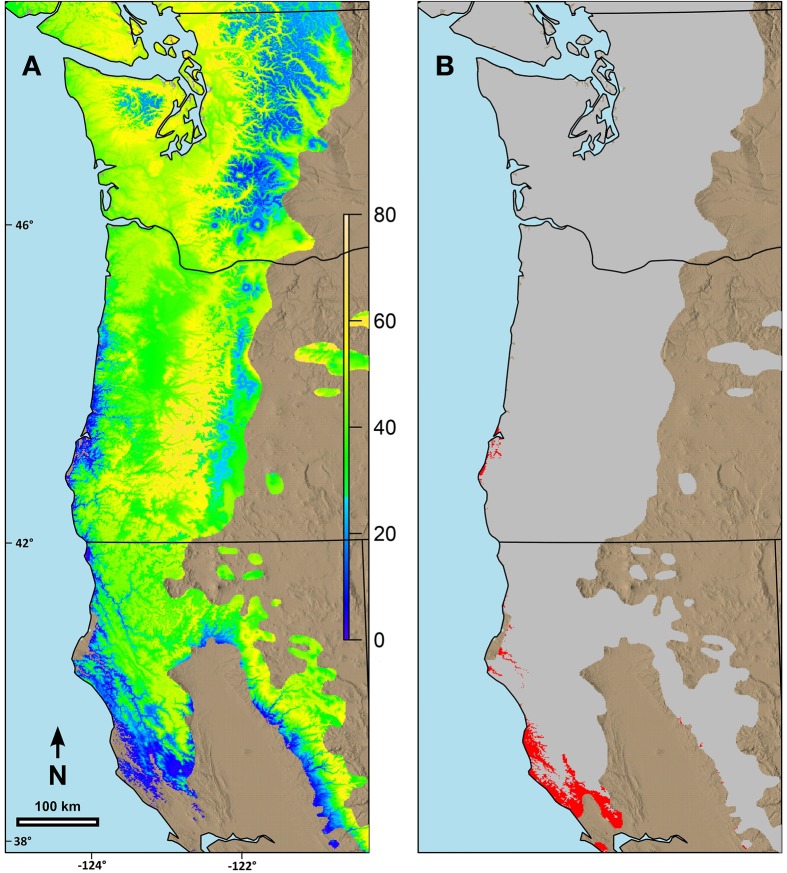
**Predicted changes in date of terminal budburst for Douglas-fir between current climatic conditions and those predicted in 2080 (consensus, A2) using ClimateWNA for the current range of the species in the study area. (A)** Sites with earlier date of budburst in 2080 than currently, **(B)** Sites with later date of budburst than currently. Under **(A)** the color scale indicates number of days earlier that budburst is predicted to occur by 2080. Under **(B)** the areas in red are where the predicted date of budburst is predicted to be later in 2080 than currently. Numbers on the left and the bottom of the map refer to latitude (north) and longitude (west).

Combining the possibility line equations for individual species or groups of species with climate information allowed prediction of date of spring budburst across broad geographic areas. Predicted date of budburst for Douglas-fir under current conditions (Figure 4A) resulted in dates which seem reasonable based on personal observations and published data (Harrington et al., 2010; Gould et al., 2011). Predicted date of budburst for Douglas-fir in 2080 (Figure 4B) was generally early in the spring, especially in low elevation areas (e.g., along the Pacific coast, in the Puget Trough and Willamette Valley). Changes in date of budburst between current climate and that predicted in 2080 (Figure 5A) revealed a more nuanced pattern with the least change in date of budburst at mid to high elevations, especially in Washington, and also little change close to the Pacific Ocean in southern Oregon and California and at low elevation sites in the most southern part of the species range. In general, mid to high elevation or higher latitude sites were predicted to experience more chilling in the future as hours currently below freezing which have no or limited effectiveness would be shifted into hours in the optimum chilling range. Thus, combining more chilling with more forcing resulted in earlier predicted dates of budburst. However, some areas along the southern Oregon coast and in California, especially near the southern edge of the species range were predicted to have a later date of budburst in 2080 than under current conditions (Figure 5B). Overall, the change in the date of budburst was predicted to vary from −74 (earlier) to +48 (later) days. Some sites near the coast of southern Oregon had predicted later dates of budburst in 2080 but generally by <10 days. However, substantial areas of the southern portion of the range in California had dates of budburst in 2080 which were later by up to 20 days. Only a very small area on the California coast (near Manchester) was predicted to have spring bud burst 30 or more days later in 2080 than under current climate conditions. We checked these predictions against data from multiple field sites which had a range of 47 days in budburst (across sites and years). The linear regression between temperature and measured date of bud burst was 8.6 days per degree C. This was very similar to the predictions by our model. Douglas-fir was the only species evaluated in this trial which was predicted to have later dates of budburst within its current range by 2080; however, there were geographic locations for other species where the predicted change date of budburst in 2080 was very small (e.g., western hemlock, 3 days; western redcedar, 7 days; and Pacific madrone, 8 days).

**Figure 6:**
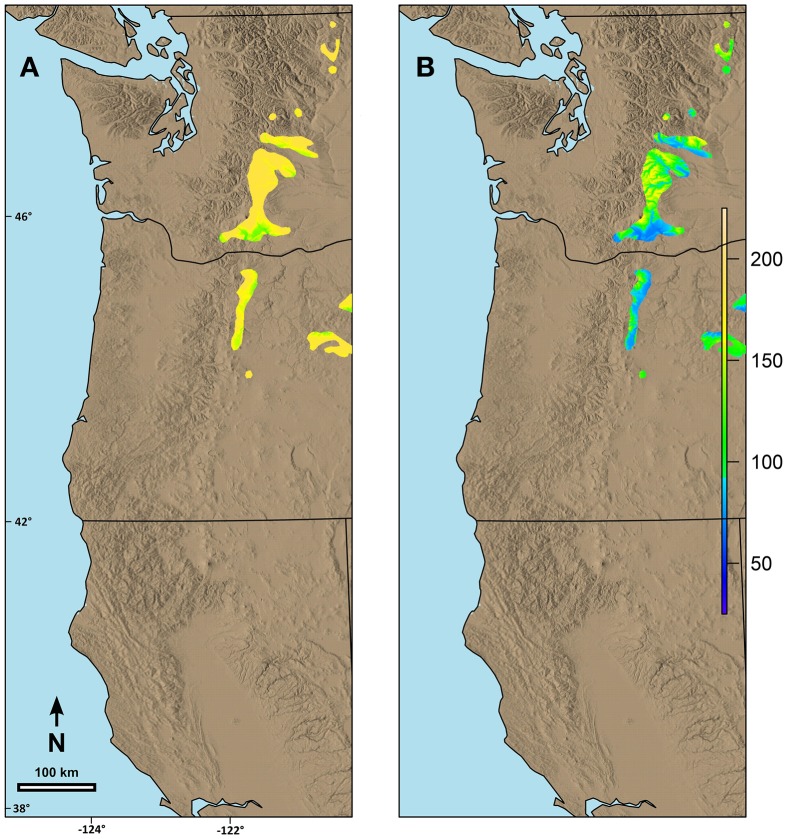
**Predicted date of terminal budburst for western larch under: (A) current climatic conditions and (B) climatic conditions in 2080 based on the A2 scenario in ClimateWNA**. The colors reflect number of days since the beginning of the year (1 = Jan 1). Numbers on the left and the bottom of the map refer to latitude (north) and longitude (west).

Predicted date of spring budburst differed by species based on both the current geographic range and the possibility line fit for the species. For example, within the area we were evaluating, the range of western larch is primarily limited to mid to high elevation areas along the eastern edge of the Cascade Range in Washington and Oregon. Predicted current date of spring budburst is generally late (Figure 6A) for these areas. By 2080, however, the date of spring budburst for western larch is predicted to advance between 17 and 84 days (Figure 6B).

**Figure 7:**
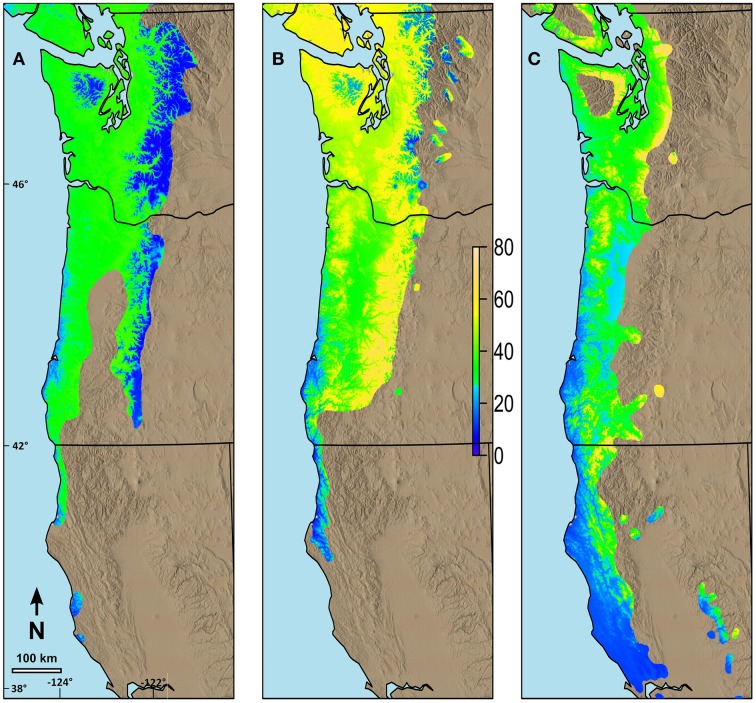
**Predicted changes in date of terminal budburst between current climatic conditions and those predicted in 2080 (consensus, A2) using ClimateWNA for the current range of the species in the study area. (A)** Changes in date of budburst for western hemlock, **(B)** Changes in date of budburst for western redcedar, **(C)** Changes in date of budburst for Pacific madrone. The color scale indicates the number of days earlier budburst is predicted to occur by 2080. Numbers on the left and the bottom of the map refer to latitude (north) and longitude (west).

Changes in date of budburst for species with long N-S ranges differed based on their current range and the differences in their possibility lines (Figure 7). We predicted relatively small changes in date of budburst by 2080 for western hemlock (Figure 7A), especially at higher elevations in the Cascade and Olympic Mountains and in areas along the southern Oregon and California coastal portions of its range. Greater advances, but generally less than 40 days, were predicted in the future date of budburst for much of the rest of the species' range in our study area. Western redcedar was predicted to have much earlier budburst by 2080 throughout most of its range (Figure 7B) with the changes greatest in the middle elevations (maximum of 72 days). Pacific madrone currently has limited distribution at mid to higher elevations. The areas predicted to have the greatest change in date of spring budburst for madrone (maximum of 76 days) were in the areas where the current climate is currently the coolest (mid elevations) (Figure 7C).

## Discussion

We earlier proposed the possibility line concept, which included modeling of budburst as a direct but non-linear tradeoff between chilling and forcing, as well as parallel accumulation of these factors, with data for Douglas-fir (Harrington et al., 2010; Gould et al., 2011). This was an extension of similar modeling work done previously for other species (Cannell and Smith, 1986; Murray et al., 1989). In this study we tested the parallel model of dormancy for several species and demonstrated it performed well under a wide range of conditions. It appeared that all the species tested in our trials benefited from additional chilling; that is, the time to budburst continued to decline. This is consistent with the argument by Landsberg (1974) that the differences in time to budburst under a range of chilling implies the buds are capable of responding to forcing when full chilling has not been achieved. Although this model of dormancy may not apply to all species, it has been observed by others for multiple species that additional chilling reduces the time to budburst under favorable conditions or reduces the amount of forcing or heat sums needed for budburst (Nelson and Lavender, 1979; Cannell and Smith, 1983; Carlson, 1985; Lavender and Stafford, 1985; Murray et al., 1989; Viherä-Aarnio et al., 2014); thus, supporting a parallel model for at least several species and conditions. In addition, budburst for two hardwood species in Arkansas occurred close to or slightly above the possibility line for Douglas-fir (Burner et al., 2014); thus, the general concept appears to be reasonable for several species in other climates.

The shape and location of the possibility lines for different species or populations can have ecological and practical interpretations. We can use the shapes of the curves to predict differences between species in their current environments and also predict how each species (and genotype) will respond if the climate changes as predicted. The possibility line for western larch is quite low at high levels of chilling; this implies very little forcing is needed to promote budburst and this is consistent with field observations that the species is the earliest one to burst bud in mixed-species stands. It was suggested that subalpine larch (*Larix lyallii*), subalpine fir (*Abies lasiocarpa*) and Pacific silver fir (*Abies amabilis*) burst bud early in the spring due to low threshold temperatures and low heat sum requirements for budburst (Worrall, 1983, 1993). Although we did not include subalpine larch or subalpine fir in our study, it is possible that those species may have differently shaped possibility lines than other species rather than different effectiveness functions for temperature. For example, subalpine larch may have a line similar to that of western larch, or perhaps even lower at high chilling, thus, very little forcing would be needed to promote budburst, i.e., it is not necessary to assume a different threshold temperature to account for what is observed naturally. Species can be grouped to illustrate their relationships between chilling and forcing as was done by Murray et al. (1989) and Cannell and Smith (1986) and these groupings can be discussed in terms of the dormancy requirements of the groups in relation to the current and projected climates. In addition, the shape of the possibility lines at low chilling will differ between species which have an obligate chilling requirement (the line has an asymptote as it will not cross the Y axis at low chilling while) and species with non-obligate chilling requirements.

Several threshold temperatures have been suggested as the upper limit for chilling efficacy in fruit trees or ornamental plants (e.g., 7.2°C for lilac in Schwartz and Hanes, 2010, 10°C by Sarvas, 1974) and many have modeled the effectiveness of chilling by assuming only temperatures less than 5°C are effective in providing chilling. We have observed normal budburst of Douglas-fir in our greenhouse when the minimum temperature was kept at 10°C so we have evidence that temperatures >10°C are effective in providing chilling to promote budburst (albeit much less effective than cooler temperatures). Extending the temperature range which is effective in satisfying chilling to temperatures greater than 5°C (and not including time at temperatures significantly below freezing) could change past predictions of how plants in specific locations will respond to altered climate.

The possibility line relationship also accounts for bud burst in cold storage in nurseries when stock has been held for long periods of time; the very high accumulation of chilling in cold (but not freezer) storage means very small amounts of forcing can promote budburst. Although high quality commercial cold storage units can maintain uniform low temperatures, variation in temperature within seedling storage bags can occur if seedling bags are stored near traffic doors, especially during periods when the doors are opened frequently. In addition, it can take several days or even longer to cool seedlings in large bags. Even very small increases in temperature may promote budburst in seedlings which have experienced large numbers of chilling units. These small increases in temperature are especially important given that forcing can accumulate anytime during the cold storage process. Under natural conditions, buds would always receive light along with chilling and forcing temperatures; however, since budburst has been observed to occur in cold storage, it appears at least for some species that light in general or specific photoperiods are not a requirement for budburst. However, for genotypes with non-obligate chilling requirements, long photoperiods can trigger budburst in the absence of chilling (e.g., Romberger, 1963; Garber, 1983).

The development of the possibility lines for individual species, groups of species or seed sources allows us to predict bud burst under future conditions predicted by climate models. Similar lines showing the tradeoff between chilling and forcing (or thermal time) were developed for groups of woody species in Scotland (Murray et al., 1989) although they used a different temperature range to define chilling than we did and their lines did not extend to very low levels of chilling. Species such as Douglas-fir with an obligate chilling requirement are likely to have range restrictions in areas which currently experience warm winters. This is consistent with earlier suggestions that warming could delay budburst if chilling was not adequate (Cannell and Smith, 1986) or result in abnormal development (Chandler et al., 1937; Weinberger, 1967). Although budburst will generally occur much earlier under the 2080 A2-emission scenario we modeled, there are areas in California and on the southern Oregon coast where the date of spring bud burst is predicted to occur later than under current climatic conditions. Later budburst could have substantial negative consequences for tree growth as less time would be available for early season shoot growth prior to the typical droughty summer conditions in these areas. Even minimal advance in the date of spring budburst could have negative consequences on tree growth and development if the timing of droughty conditions advances faster than the timing of budburst. In addition, we assume that the current range of Douglas-fir (and other species) is influenced in part by the presence of adequate chilling. For example, when Douglas-fir was planted to establish a seed orchard south of its native range near Monterey in central California, the planting was deemed a failure due to poor growth, non-elongation of terminal buds and increased mortality (Copes, 1983); these results were attributed to lack of chilling. Thus, selection of species and appropriate genotypes for future plantings should consider temperatures during the dormant season as well as other factors.

Predicting changes in date of terminal budburst between current conditions and 2080 can indicate which species will exhibit the greatest changes and where those changes will occur. For species where we predicted a substantial change in number of days to budburst between current and 2080 climates (e.g., Douglas-fir, western larch, Pacific madrone, and western redcedar) the greatest effect of climate warming was predicted to occur at mid to high elevations. On the other hand, as was suggested by Cannell and Smith (1986) warming temperatures have little effect on advancing budburst for species with obligate chilling requirements if the current conditions during the dormant season are currently warm (providing little effective chilling). These trees will not be able to significantly advance their date of budburst to take advantage of suitable environmental conditions for shoot growth if their dormancy requirement has not been met.

Some of the extreme values for date of budburst are a side-effect of the way in which our species range maps were used. That is, our projections assumed all areas within the boundary of the range maps were suitable for growth of the species when in fact some areas such as high elevation alpine environments are not suitable for growth of our tree species. In addition, our budburst models are based on seedlings and many characteristics associated with growth are expressed more conservatively in older trees (Kramer and Kozlowski, 1979). The reason for observed differences in timing of budburst with plant age have not been well studied and may reflect differences in microclimate for buds on plants of different sizes/ages as well as differences in physiological responses to environmental stimuli. Thus, our predictions for date of spring budburst may be more extreme than will occur under a changed climate.

The original possibility line calculations assumed that the accumulation of chilling and forcing began November 1 and accumulation of chilling ended March 21 (Harrington et al., 2010). This worked well on mild sites where date of budburst occurred early in the spring or in our experiments where the use of greenhouses and growth chambers resulted in early budburst. However, we later realized that we would have better fits of our possibility lines at high levels of chilling if we did not stop accumulating chilling at that date. We have since eliminated the March 21 cut-off date so that in the current analyses chilling units, as well as forcing, continue to accumulate until bud burst. It was suggested by Rinne et al. (2011) that chilling was complete in 8 weeks; this is not consistent with our observation that even at high levels of chilling, additional chilling appears to be beneficial in reducing the amount of forcing needed for bud burst. In addition, many others have demonstrated substantial additional benefit in chilling beyond 8 weeks (c.f., Nelson and Lavender, 1979; Cannell and Smith, 1983). We do not know if this difference in the optimum level of chilling is due to a difference in response between plants adapted to boreal or more temperate environments or if other factors may be involved. The date at which chilling and forcing should begin to accumulate presumably should depend on when buds become truly dormant in the fall. Thus, the timing of when chilling and forcing units should accumulate should roughly correspond to some time period after a winter bud is set. For our Pacific Northwest environment, calculations with different initial dates to begin accumulation of chilling and forcing did not result in much change in the fit of the possibility lines. We selected November 1 somewhat arbitrarily based on our climate but expect the appropriate date would probably differ somewhat depending on species and environmental conditions. It can be difficult to determine the starting date to begin dormancy calculations from past trials as the environmental data in different time periods is highly correlated. It may be more productive to conduct trials to determine when true dormancy occurs or to base the starting time for calculations on a set number of weeks after a well-developed winter bud was set (or based on anatomical criteria such as the number of rows of leaf primordia which have been initiated).

Modeling the date of budburst is hampered by our lack of knowledge as to the physiological mechanisms involved. Past studies have demonstrated that plants are responding to air rather than soil temperature and it has been inferred that buds are the tissues which experience the environment. Thus, most studies related to chilling for fruit trees are done on twigs moved into greenhouses or other warm environments. However, species without vegetative buds such as redcedar can also have chilling requirements; that is, they require fewer forcing units if they experience chilling temperatures, so we can infer that it is the shoot apical meristem which is involved in dormancy. In addition, it has long been observed in *Pinus* that stem elongation and needle development can have separate kinetics—that is, stem elongation can begin long before needle elongation occurs. The shoot apical meristem is composed of: a peripheral zone, which creates leaves, cones, and flowers; a rib zone, which creates pith and stem; and a central zone which replenishes the peripheral and rib zones (Sharma and Fletcher, 2002). Based on our observed responses in plant development, it could be hypothesized that in species such as Douglas-fir, true fir or madrone, the peripheral and rib zones are responding at the same time and in the same way to winter dormancy cues while in pines, the rib zone may respond earlier and differentially than the peripheral zone.

Except for western hemlock, the species evaluated in this trial which demonstrated obligate chilling requirements exhibited abnormal morphology without chilling. It has been long observed that insufficiently chilled plants may grow abnormally (Romberger, 1963). In addition, the observation that dwarfing or lack of internodal elongation was more pronounced in the main epicotyledonary axis (Tukey and Carlson, 1945) is consistent with our observations that on some plants lateral buds may burst normally or that the shoot apical meristem may have increased in size sufficiently to expose the needles in the buds but additional development (stem division) does not occur. Of course, species such as redcedar which do not form vegetative buds do have shoot apical meristems and these meristems appear to respond quickly with new growth when the plants are in favorable environments (Grossnickle and Russell, 2006). However, more work is needed to determine why the redcedar plants responded differently in 2012 and 2013, in particular, we need to understand which factors influence future growth responses of this species. One possibility is the plants in 2013 were not dormant in the fall and thus did not respond to chilling.

Both our temperature monitoring and model development used air temperature as a surrogate for temperature in the shoot apical meristem. Meristem (or bud) temperatures can be warmer or colder than air temperature depending on physical and biological factors (Grace et al., 1989; Apple et al., 1999; Grace, 2006; Savvides et al., 2013). Monitoring temperature in the meristems is important for mechanistic studies of plant development. For example, monitoring meristem temperature will help clarify if temperatures slightly below freezing have any effectiveness in satisfying chilling, as our model of effectiveness predicts, or if the apparent effectiveness is an artifact of the way temperatures are usually measured (i.e., measurement of air in a shaded shelter rather than within plant tissues). On the other hand, models which predict the effects of future temperatures on dormancy have many potential sources of error, and the location from which meristem temperature is measured or predicted is just one source. In addition, models of future climate will predict air temperature and not temperature at specific plant tissues.

Many studies have modeled budburst in trees as a function of temperature (c.f., Cannell and Smith, 1983; Fu et al., 2012). Some have concluded that the most effective predictive models are those which do not consider chilling (Fu et al., 2012); however, those results can be interpreted as implying that the data being used in modeling comes from the right hand side of the possibility line where plants have experienced adequate chilling. Models which do not consider chilling are not likely to be useful in predicting responses in future warmer climates which will include winter temperatures outside the range of past conditions (IPCC, 2013).

The lack of normal budburst in seedlings of many species kept in the greenhouse during the winter along with the observed changes in seedling form (minimal new leaf area, change in terminal leader, low growth rates) under low chilling indicate that warmer winter temperatures may have much greater impacts on Pacific Northwest forests than most managers or policy makers are currently considering. On the other hand, our results imply that some species such as western redcedar and Pacific madrone may be better adapted than some of their forest associates to warmer winter temperatures. Although the information is based on only a few genotypes, the pine genotypes evaluated had similar observed responses. However, due to differences in the responses among pine species at low chilling and reported differences in responses to chilling or lack of chilling previously reported for ponderosa pine (Burr et al., 1989; Omi et al., 1991; Sloan, 1991; Wenny et al., 2002) we plan to follow up with trials involving more genotypes per pine species. Douglas-fir (Gould et al., 2011) and Pacific madrone (this study) both exhibited substantial differences in the possibility lines for genotypes within each species, indicating substantial genetic variation may exist in chilling and forcing requirements for budburst. Understanding this type of variation could allow managers to select genotypes with chilling and forcing requirements better matched to future climatic conditions. Species with non-obligate chilling requirements (such as loblolly pine) may burst bud as day length increases in the spring in mid-latitudes (Garber, 1983); however, at low latitudes such as in the Hawaiian Islands, day length does not vary sufficiently to compensate for lack of chilling (Carlson and Harrington, 1995). Thus, responses to warmer winter temperatures may vary substantially with genotype as well as with multiple environmental factors.

Current knowledge appears adequate to predict that the timing of future bud burst will change substantially over time and in some areas the predicted climate conditions will not result in normal bud burst. In mid to upper northern latitudes this is most likely to occur at the southern and coastal edges of species' ranges where current winter conditions are mild. Areas currently experiencing cold winters (e.g., high elevation or high latitudes) will experience greater chilling as winter warming will convert hours in sub-freezing temperatures with no or limited effectiveness in satisfying chilling to hours slightly above freezing temperatures with high chilling effectiveness; this is likely to advance the timing of spring budburst but it is not yet clear if this is likely to result in greater likelihood of spring frost damage (Hänninen, 2006). Tree breeding or selection of specific genotypes with lower chilling requirements (lower possibility lines at low levels of chilling) will probably be useful in mitigating the effects of warmer winters in many areas but scientific breakthroughs or selection of species with non-obligate chilling requirements may be required for timber production in areas which currently experience winters with little chilling.

### Conflict of interest statement

The authors declare that the research was conducted in the absence of any commercial or financial relationships that could be construed as a potential conflict of interest.

## References

[B1] Ahas R. Aasa A. Menzel A. Fedotova V. G. Scheifinger H. (2002). Changes in European spring phenology. Int. J. Clim. 22, 1727–1738 10.1002/joc.818

[B2] Apple M. E. Lucash M. S. Phillips D. L. Olszyk D. M. Tingey D. T. (1999). Internal temperature of Douglas-fir buds is altered at elevated temperature. Environ. Exp. Bot. 41, 25–30 10.1016/S0098-8472(98)00046-X

[B3] Bates D. M. Watts D. G. (1988). Nonlinear Regression Analysis and Its Applications. Hoboken, NJ: John Wiley & Sons, Inc 10.1002/9780470316757

[B4] Burner D. M. Brauer D. K. Snider J. L. Harrington C. A. Moore P. A. Jr. (2014). Phenological responses of juvenile pecan and white oak on an upland site. Agroforest. Syst. 88, 141–155 10.1007/s10457-013-9662-5

[B5] Burr K. E. Tinus R. W. Wallner S. J. King R. M. (1989). Relationships among cold hardiness, root growth potential and bud dormancy in three conifers. Tree Physiol. 5, 291–306. 10.1093/treephys/5.3.291 14972975

[B6] Calmé S. Bigras F. J. Margolis H. A. Hébert C. (1994). Frost tolerance and bud dormancy of container-grown yellow birch, red oak and sugar maple seedlings. Tree Physiol. 14, 1313–1325. 10.1093/treephys/14.12.1313 14967606

[B7] Campoy J. A. Ruiz D. Egea J. (2011). Dormancy in temperate fruit trees in a global warming context: a review. Sci. Hortic. 130, 357–372 10.1016/j.scienta.2011.07.011

[B8] Cannell M. G. R. Smith R. I. (1983). Thermal time, chill days and prediction of budburst in *Picea sitchensis*. J. Appl. Ecol. 20, 951–963 10.2307/2403139

[B9] Cannell M. G. R. Smith R. I. (1986). Climatic warming, spring budburst, and forest damage on trees. J. Appl. Ecol. 23, 177–191. 10.2307/240309012651493

[B10] Carlson W. C. (1985). Effects of natural chilling and cold storage on budbreak and root growth potential of loblolly pine (*Pinus taeda* L.). Can. J. For. Res. 15, 651–656 10.1139/x85-106

[B11] Carlson W. C. Harrington C. A. (1995). Effects of winter temperatures on growth and apical dominance of *Pinus taeda*. Plant Physiol. 108, 115.7784501

[B12] Chandler W. H. Kimball M. H. Phillip L. Tufts W. P. Weldon G. P. (1937). Chilling Requirements for Opening of Buds on Deciduous Orchard Trees and Some Other Plants in California. Berkeley, CA: University of California, College of Agriculture, Agricultural Experimental Station.

[B13] Copes D. L. (1983). Failure of grafted Douglas-fir planted at Monterey, CA. Tree Planters' Notes 34, 9–10.

[B14] Cumming S. R. Burton P. J. (1996). Phenology-mediated effects of climatic change on some simulated British Columbia forests. Clim. Change 34, 213–222 10.1007/BF00224632

[B15] Fu Y. H. Campioli M. Van Oijen M. Deckmyn G. Janssens I. A. (2012). Bayseian comparison of six different temperature-based budburst models for four temperature tree species. Ecol. Model. 230, 92–100 10.1016/j.ecolmodel.2012.01.010

[B16] Garber M. P. (1983). Effects of chilling and photoperiod on dormancy release of container-grown loblolly pine seedlings. Can. J. For. Res. 13, 1265–1270. 10.1139/x83-16914972934

[B17] Gould P. J. Harrington C. A. St Clair J. B. (2011). Incorporating genetic variation into a model of budburst phenology of coast Douglas-fir (*Pseudotsuga menziesii var. menziesii*). Can. J. For. Res. 41, 139–150 10.1139/X10-191

[B18] Grace J. (2006). The temperature of buds may be higher than you thought. New Phytol. 170, 1–3. 10.1111/j.1469-8137.2006.01675.x 16539596

[B19] Grace J. Allen S. Wilson C. (1989). Climate and meristem temperatures of plant communities near the tree-line. Oecologia 79, 198–204 10.1007/BF00388479 28312856

[B20] Grossnickle S. C. Russell J. H. (2006). Yellow-cedar and western redcedar ecophysiological response to fall, winter, and early spring temperature conditions. Ann. For. Sci. 63, 1–8 10.1051/forest:2005092

[B21] Hänninen H. (1995). Effects of climatic change on trees from cool and temperate regions; an ecophysiological approach to modeling of bud burst phenology. Can. J. Bot. 73, 183–199 10.1139/b95-022

[B22] Hänninen H. (2006). Climate warming and the risk of frost damage to boreal forest trees: identification of critical ecophysiological traits. Tree Physiol. 26, 889–898. 10.1093/treephys/26.7.889 16585034

[B23] Hänninen H. Kramer K. (2007). A framework for modelling the annual cycle of trees in boreal and temperate regions. Silva Fennica 41, 167–205 10.14214/sf.313

[B24] Harrington C. A. Gould P. J. St Clair J. B. (2010). Modeling the effects of winter environment on dormancy release of Douglas-fir. For. Ecol. Manag. 259, 798–808 10.1016/j.foreco.2009.06.018

[B25] IPCC. (2013). Climate change 2013: the physical science basis, in Contribution of Working Group I to the Fifth Assessment Report of the Intergovernmental Panel on Climate Change, eds Stocker T. F. Qin D. Plattner G.-K. Tignor M. Allen S. K. Boschung J. Nauels A. Xia Y. Bex V. Midgley P. M. (Cambridge; New York: Cambridge University Press). Available online at: http://www.ipcc.ch/report/ar5/wg1/

[B26] Jeong S.-J. Ho C.-H. Gim H.-J. Brown M. E. (2011). Phenology shifts at start vs. end of growing season in temperate vegetation over the Northern Hemisphere for the period 1982–2008. Glob. Change Biol. 17, 2385–2399 10.1111/j.1365-2486.2011.02397.x

[B27] Kramer K. (1994). Selecting a model to predict the onset of growth of *Fagus sylvatica*. J. Appl. Ecol. 31, 172–181 10.2307/2404609

[B28] Kramer P. J. Kozlowski T. T. (1979). Physiology of Woody Plants. New York, NY: Academic Press.

[B29] Landsberg J. J. (1974). Apple fruit bud development and growth: analysis and an empirical model. Ann. Bot. 38, 1013–1023.

[B30] Lavender D. P. Stafford S. G. (1985). Douglas-fir seedlings: some factors affecting chilling requirement, bud activity, and new foliage production. Can. J. For. Res. 15, 309–312 10.1139/x85-050

[B31] Leida C. Terol J. Martí G. Agustí M. Llácer G. Badenes M. L.. (2010). Identification of genes associated with bud dormancy release in Prunus persica by suppression subtractive hybridization. Tree Physiol. 30, 655–666. 10.1093/treephys/tpq008 20231169

[B32] Molisch H. (1906). Über ein einfaches Verfahren, Pflanzen zu treiben. Sitzber. Akad. Wiss. 118, 637–690.

[B33] Murray M. B. Cannell M. G. R. Smith R. I. (1989). Date of budburst of fifteen tree species in Britain following climatic warming. J. Appl. Ecol. 26, 693–700 10.2307/2404093

[B34] Murray M. B. Smith R. I. Leith I. D. Fowler D. Lee H. S. J. Friend A. D.. (1994). Effects of elevated CO_2_, nutrition and climate warming on bud phenology in Sitka spruce (*Picea sitchensis*) and their impact on the risk of frost damage. Tree Physiol. 14, 691–706. 10.1093/treephys/14.7-8-9.691 14967641

[B35] Nelson E. A. Lavender D. P (1979). The chilling requirement of western hemlock seedlings. For. Sci. 25, 485–490.

[B36] Nychka D. Furrer R. Sain S. (2014). Fields: Tools for Spatial Data. R package version 7.1. Available online at: http://CRAN.R-project.org/package=fields

[B37] Omi S. K. Rose R. Sabin T. E. (1991). Effectiveness of freezer storage in fulfilling the chilling requirement of fall-lifted ponderosa pine seedlings. New Forests 5, 307–326.

[B38] R Core Team. (2013). R: A Language and Environment for Statistical Computing. Vienna: R Foundation for Statistical Computing Available online at: http://www.R-project.org/

[B39] Rinne P. L. H. Kaikuranta P. M. van der Schoot C. (2001). The shoot apical meristem restores its symplasmic organization during chilling-induced release from dormancy. Plant J. 26, 249–264. 10.1046/j.1365-313X.2001.01022.x 11439114

[B40] Rinne P. L. H. Welling A. Vahala J. Ripel L. Ruonala R. Kangasjärvi J.. (2011). Chilling of dormant buds hyperinduces *FLOWERING LOCUS T* and recruits GA-inducible 1,3-β-glucanases to reopen signal conduits and release dormancy in *Populus*. Plant Cell 23, 130–146. 10.1105/tpc.110.081307 21282527 PMC3051240

[B41] Rohde A. Bhalero R. P. (2007). Plant dormancy in the perennial context. Trends Plant Sci. 12, 217–223. 10.1016/j.tplants.2007.03.012 17416545

[B42] Romberger J. A. (1963). Meristems, Growth, and Development in Woody Plants: An Analytical Review of Anatomical, Physiological, and Morphogenic Aspects. Washington, DC: U.S. Goverment printing office

[B43] Sarvas R. (1974). Investigations on the annual cycle of development of forest trees. II Autumn dormancy and winter dormancy. Commun. Inst. For. Fenn. 84, 1–101.

[B44] Saure M. C. (1985). Dormancy release in deciduous fruit trees. Hort. Rev. 7, 239–300 10.1051/forest:2005092

[B45] Savvides A. van Ieperen W. Dieleman J. A. Marcelis L. F. M. (2013). Meristem temperature substantially deviates from air temperature even in moderate environments; is the magnitude of this deviation species specific? Plant Cell Environ. 36, 1950–1960. 10.1111/pce.12101 23509944

[B46] Schwartz M. D. Hanes J. M. (2010). Continental-scale phenology: warming and chilling. Int. J. Clim. 30, 1595–1598. 10.1002/joc.201422610120

[B47] Sharma V. K. Fletcher J. C. (2002). Maintenance of shoot and floral meristem cell proliferation and fate. Plant Physiol. 129, 31–39. 10.1104/pp.010987 12011335 PMC1540224

[B48] Sloan J. P. (1991). Ponderosa and Lodgepole Pine Seedling Bud Burst Varies with Lift Date and Cultural Practices in an Idaho Nursery. Res. Note. INT-397. Ogden, UT: USDA For. Serv. Res.

[B49] Sung S. Amasino R. M. (2005). Remembering winter: toward a molecular understanding of vernalization. Ann. Rev. Plant Biol. 56, 491–508. 10.1146/annurev.arplant.56.032604.144307 15862105

[B50] Tukey H. B. Carlson R. F. (1945). Morphological changes in peach seedlings following after-ripening treatments of the seeds. Bot. Gaz. 106, 431–440 10.1086/335315

[B51] Ubi B. E. Sakamoto D. Ban Y. Shimada T. Ito A. Nakajima I. (2010). Molecular cloning of dormancy-associated MADS-box gene homologs and their characterization during seasonal endodormancy transitional phases of Japanese pear. J. Am. Soc. Hortic. Sci. 135, 174–182.

[B52] van den Driessche R. (1977). Survival of coastal and interior Douglas fir seedlings after storage at different temperatures, and effectiveness of cold storage in satisfying chilling requirements. Can. J. For. Res. 7, 125–131 10.1139/x77-018

[B53] Viherä-Aarnio A. Sutinen S. Partanen J. Häkkinen R. (2014). Internal development of vegetative buds of Norway spruce trees in relation to accumulated chilling and forcing temperatures. Tree Physiol. 34, 547–556. 10.1093/treephys/tpu038 24876293

[B54] Wang T. Hamann A. Spittlehouse D. Murdock T. N. (2012). ClimateWNA - High-resolution spatial climate data for western North America. J. Appl. Meteorol. Clim. 61, 16–29 10.1175/JAMC-D-11-043.1

[B55] Weinberger J. H. (1967). Some temperature relations in the natural breaking of the rest of peach flower buds in the San Joaquin Valley, California. J. Am. Soc. Hortic. Sci. 91, 84–89 10.1051/forest:2005092

[B56] Wenny D. L. Swanson D. J. Dumroese R. K. (2002). The chilling optimum of Idaho and Arizona ponderosa pine buds. West. J. Appl. For. 17, 117–121.

[B57] Worrall J. (1983). Temperature – bud-burst relationships in amabilis and subalpine fir provenance tests replicated at different elevations. Silvae Genet. 32, 203–209.

[B58] Worrall J. (1993). Temperature effects on bud-burst and leaf-fall in subalpine larch. J. Sustain. For. 1, 1–18 10.1300/J091v01n02_01

